# Elective induction for pregnancies at or beyond 41 weeks of gestation and its impact on stillbirths: a systematic review with meta-analysis

**DOI:** 10.1186/1471-2458-11-S3-S5

**Published:** 2011-04-13

**Authors:** Arwa Abbas Hussain, Mohammad Yawar Yakoob, Aamer Imdad, Zulfiqar A Bhutta

**Affiliations:** 1 Division of Women and Child Health, The Aga Khan University, Stadium Road, P.O. Box 3500, Karachi, Pakistan

## Abstract

**Background:**

An important determinant of pregnancy outcome is the timely onset of labor and birth. Prolonged gestation complicates 5% to 10% of all pregnancies and confers increased risk to both the fetus and mother. The purpose of this review was to study the possible impact of induction of labour (IOL) for post-term pregnancies compared to expectant management on stillbirths.

**Methods:**

A systematic review of the published studies including randomized controlled trials, quasi- randomized trials and observational studies was conducted. Search engines used were PubMed, the Cochrane Library, the WHO regional databases and hand search of bibliographies. A standardized data abstraction sheet was used. Recommendations have been made for input to the Lives Saved Tool (LiST) model by following standardized guidelines developed by the Child Health Epidemiology Reference Group (CHERG).

**Results:**

A total of 25 studies were included in this review. Meta-analysis of 14 randomized controlled trials (RCTs) suggests that a policy of elective IOL for pregnancies at or beyond 41 weeks is associated with significantly fewer perinatal deaths (RR=0.31; 95% CI: 0.11-0.88) compared to expectant management, but no significant difference in the incidence of stillbirth (RR= 0.29; 95% CI: 0.06-1.38) was noted. The included trials evaluating this intervention were small, with few events in the intervention and control group. There was significant decrease in incidence of neonatal morbidity from meconium aspiration (RR = 0.43, 95% CI 0.23-0.79) and macrosomia (RR = 0.72; 95% CI: 0.54 – 0.98). Using CHERG rules, we recommended 69% reduction as a point estimate for the risk of stillbirth with IOL for prolonged gestation (> 41 weeks).

**Conclusions:**

Induction of labour appears to be an effective way of reducing perinatal morbidity and mortality associated with post-term pregnancies. It should be offered to women with post-term pregnancies after discussing the benefits and risks of induction of labor.

## Background

An important determinant of the pregnancy outcome is the timely onset of labor and birth. Both preterm and post-term births are associated with unfavorable maternal and neonatal outcomes. Prolonged gestation complicates 5% to 10% of all pregnancies and confers increased risk to both the fetus and mother [1,2]. In the United States, about 18% of all singleton pregnancies persist beyond 41 weeks, 10% (range, 3% to 14%) continue beyond 42 weeks and 4% (range, 2% to 7%) continue beyond 43 completed weeks in the absence of an obstetric intervention [2,3]. Post-term pregnancy is associated with higher rates of stillbirth, macrosomia (birth weight >4000gm), birth injury and meconium aspiration syndrome [2]. The major cause of perinatal morbidity and mortality in post-term pregnancy is presumed to be the progressive uteroplacental insufficiency [4,5].

Many studies have assessed the gestation-specific stillbirth rate which is expressed as the number of stillbirths per 1000 total births at each week of gestation. Divon and colleagues [6] conducted a retrospective analysis of all deliveries in Sweden from 1987 to 1992. They found a statistically significant increase in the odds ratio for fetal death from 41 weeks and beyond. Using fetal mortality at 40 weeks' gestation as a reference level, the odds ratios for fetal death were 1.5, 1.8, and 2.9 at 41, 42, and 43 weeks, respectively. Perinatal mortality (defined as stillbirths plus early neonatal deaths) at 42 weeks of gestation was twice that at 40 weeks (4 to 7 vs. 2 to 3 per 1000 deliveries, respectively) and increases 4-fold at 43 weeks and 5- to 7-fold at 44 weeks [3,7-9].

Currently there are no tests available to ascertain whether it would be better to continue with the pregnancy or to induce birth, or tests that can determine the best possible time for induction [10]. The Society of Obstetricians and Gynecologists of Canada Clinical Practice Guidelines proposed induction of labour between 41 and 42 weeks of gestation [11]. Previously some authors believed that the policy of IOL between 41-42 weeks was a crude approach for reducing stillbirth rates because even though the risk of fetal death is increased post-term, many more fetal deaths occur between 37 and 42 weeks than do so beyond 42 weeks [12,13]. However available evidence from clinical trials and systematic reviews do suggest an impact of induction of labour on perinatal mortality. [14].

The purpose of this review was to assess the impact of elective induction of labour for post-term pregnancies (> 41 weeks) of gestation on stillbirths compared to expectant management (policy of awaiting spontaneous onset of labour). This paper is part of a series of papers which seek to estimate effect of an intervention for input into the Lives Saved Tool (LiST) model [15]. An intervention is currently included in the Lives Saved Tool (LiST) model if there is evidence that it reduces maternal mortality, infant/child mortality (<5 years) and/or stillbirths. The process of generating recommendations for an intervention involve qualitative evaluation of available evidence according to adapted GRADE criteria [16] and quantitative evaluation according to Child Health Epidemiology Reference Group (CHERG) rules [15]. For more details of the review methods, the adapted GRADE approach, see the methods section and the CHERG method paper [15].

## Methods

### Literature search

**Figure 1:**
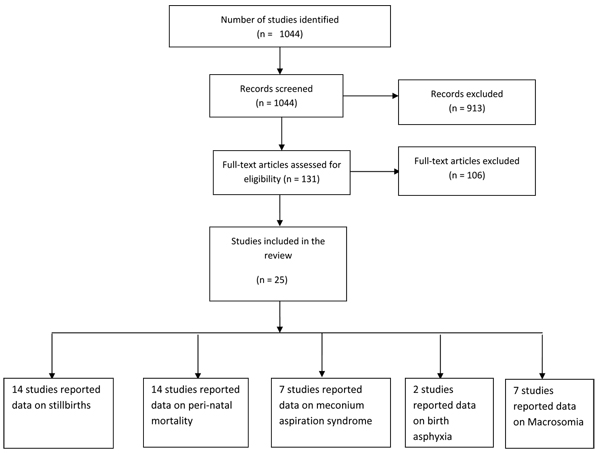
Flow diagram showing identification of studies evaluating induction of labor at or beyond 41 weeks

A literature search was conducted using search engines like PubMed, the Cochrane Library and the WHO regional databases. We identified all studies that compared the impact of elective induction of labour versus expectant management, for pregnancies at or beyond 41weeks of gestation, on stillbirths and perinatal mortality (figure 1). Furthermore, hand search of bibliographies of relevant reviews was performed. Experts in the field were contacted for further data or for unpublished trials. We also looked at previous reviews for studies that reported outcomes on morbidities associated with prolonged pregnancy. Morbidities considered in our review include meconium aspiration syndrome, birth asphyxia, and macrosomia. The following search strategy was used:

("Pregnancy, Prolonged"[Mesh] OR "Labor, Induced"[Mesh] OR induction) AND ("Labor, Obstetric"[Mesh] OR labor) AND ("Stillbirth"[Mesh] OR "Perinatal Mortality"[Mesh] OR "Fetal Death"[Mesh] OR Stillbirth* OR "Intrauterine death*" OR "perinatal mortality").

For the initial selection, there was no restriction with respect to the language of the article. However, non-English articles were not translated and if the desired information was available in the abstracts then those were used. If full texts could not be retrieved relevant available information was used from the abstract.

### Inclusion criteria

•	All studies that were included in our review looked at the impact of elective induction of labour for pregnancies at or beyond 41 weeks on stillbirths or perinatal mortality.

•	The study designs selected were randomized controlled trials, quasi experimental studies and observational studies.

•	The eligible studies were those that compared the impact of labour induction with expectant management.

•	Only those studies were included that evaluated low risk pregnancies i.e. uncomplicated, singleton, and live pregnancies at or beyond 41 completed weeks of gestation.

•	Selected studies clearly documented the outcome data on stillbirth and/or perinatal mortality.

•	The selected studies could differ in terms of the methods of induction used and/or the techniques used for monitoring of pregnancies that were managed expectantly.

•	Only data from RCTs were included in the meta-analyses.

### Exclusion criteria

•	Studies investigating the impact of induction of labour prior to 41 completed weeks on stillbirth and/or perinatal mortality were excluded.

•	Studies that looked at induction of labour for reasons other than prolonged gestation such as macrosomia etc. were excluded.

•	Studies that included complicated or high-risk pregnancies were excluded.

•	Studies comparing different methods of labour induction (means no control group with expectant management) or those comparing induction at 41 weeks with induction at 42 weeks were excluded.

### Validity assessment

We graded the ‘overall’ quality of evidence of an outcome according to the CHERG adaptation of the GRADE technique [16]. This technique is based on the following components 1) the volume and consistency of the evidence; 2) precision of the effect size or risk ratio; and 3) the level of statistical evidence for an association between the intervention and outcome, as reflected by the p-value [15,16]. The individual studies were also graded into ‘high’, ‘moderate’, ‘low’ and ‘very low’. Studies were graded ‘high’ if it were a randomized or cluster randomized trial. The grade was then decreased by one for each study design limitation. The grade was increased by 1-2 for studies reporting a statistically significant level of impact (>80% reduction) or an intent-to-treat analysis. A study with a final grade of very low was excluded based on inadequate quality. For more details of the review methods, the adapted GRADE approach or the LiST model see other articles in this supplement.

### Data abstraction

Two researchers’ extracted data into a standard web excel sheet designed by the CHERG/LiST review group [15]. The variables included were, for example, location and setting of the study, its design and limitations, allocation concealment, blinding assessment, rates of lost to follow-up, intention-to-treat analysis, definitions of intervention and control group.

#### Quantitative data synthesis

The study design considered for meta-analyses included randomized controlled trials only. Meta-analyses were generated using Generic Inverse Variance method of meta-analysis [17]. Quasi-randomized trials and observational studies were also considered for this review, but not included in meta-analyses. The definitions of stillbirth and perinatal mortality were taken as defined by the author(s). Summary estimates were described as risk ratios along with 95% confidence intervals. Fixed models were used for primarily analyses. Statistical heterogeneity among trials was assessed by observing the overlap of the confidence intervals among the studies, Chi square value (P-value) of heterogeneity I^2^ statistics. A p value > 0.1 or I^2^ value > 50% was taken to represent substantial heterogeneity and random models were used [18].

## Results

### Trial flow

The search strategy identified a total of 1044 records (Fig 1). After initial screening of the titles and abstracts, we selected 131 papers for data on the outcome measures of interest. A total of 25 relevant papers have been included in the final database. The pertinent characteristics of all included studies are given in Additional files 1, 2 and 3. The detailed data extraction with the limitation of studies is shown in Additional File 4.

### Results of the meta-analyses

**Figure 2:**
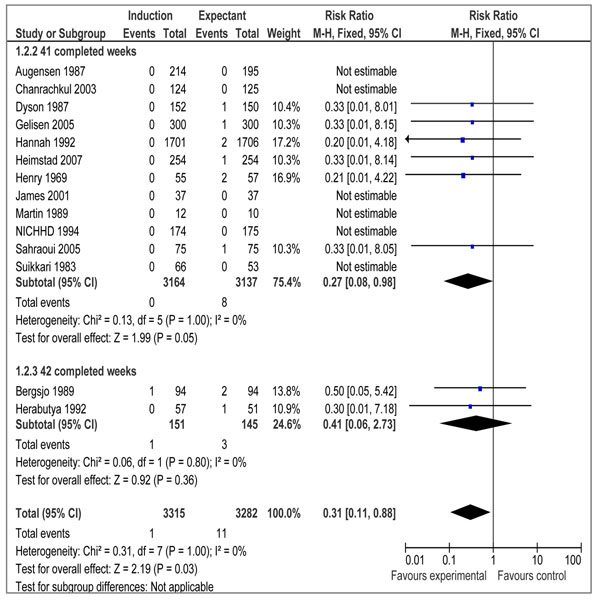
Induction of labour versus expectant management; Outcome: perinatal death

**Figure 3:**
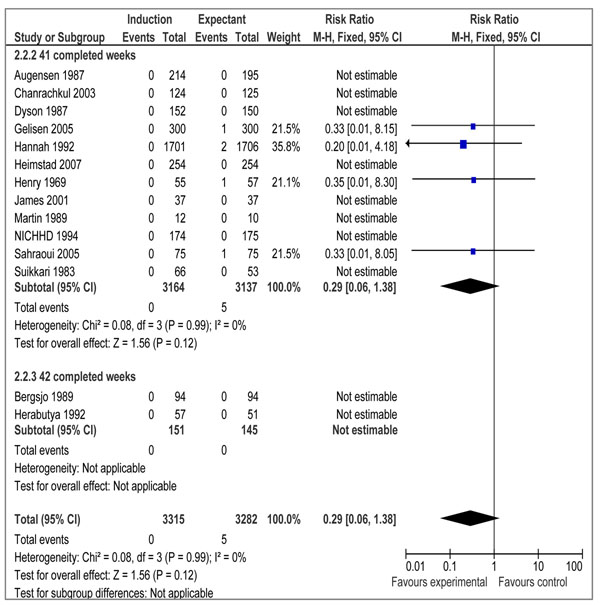
Induction of labour versus expectant management; Outcome: stillbirth

There were 16 RCTs [19-34] and 3 quasi-experimental studies [35-37] reporting outcome measures of interest. Our meta-analyses of RCTs demonstrated that elective IOL for post-term pregnancies at or beyond 41 weeks, has a statistically significant impact on reducing perinatal mortality (RR=0.31 95% CI 0.11-0.88), but the impact on stillbirth failed to reach a level of statistical significance (RR= 0.29; 95% CI: 0.06-1.38) (figures 2 and 3 respectively). We also calculated number needed to treat (NNT) for perinatal mortality (328 to reduce one case) and stillbirths (657 to avoid one stillbirth). A subgroup analysis for IOL at 41 completed weeks (based on 12 RCTs) showed a statistically significant impact on perinatal mortality (RR=0.27 95% CI 0.08-0.98), while the impact on stillbirth remained statistically insignificant (RR=0.29 95%CI 0.06-1.38) (data not shown). The results of IOL at 42 weeks based on 2 RCTs showed a statistically insignificant impact on perinatal mortality (RR=0.41 95% CI 0.06-2.73), while the impact on stillbirth was not estimable since both the studies reported zero estimates for stillbirth.

**Figure 4:**
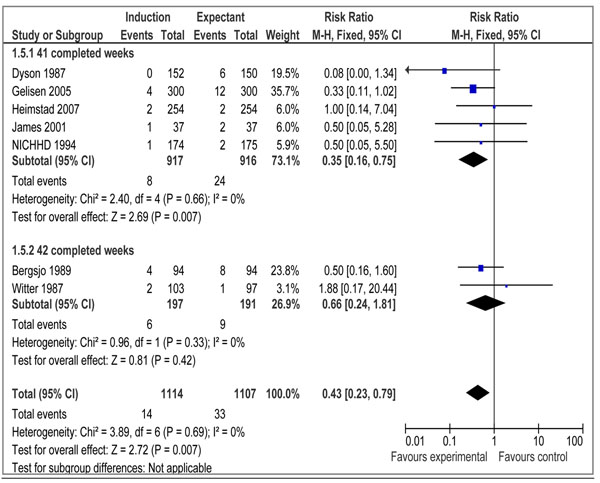
Induction of labour versus expectant management; Outcome: Meconium Aspiration Syndrome

**Figure 5:**
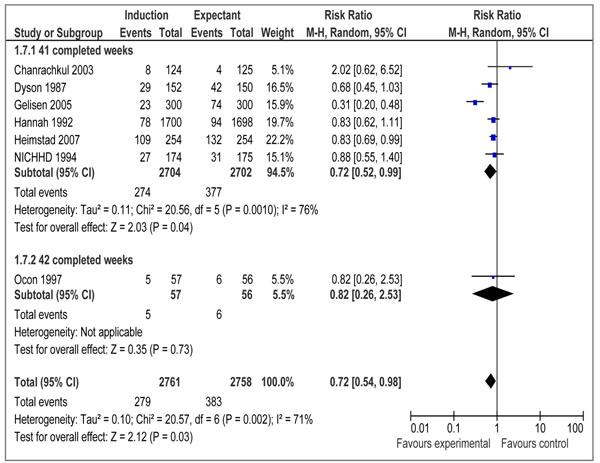
Induction of labour versus expectant management; Outcome: macrosomia (birth weight > 4000gm)

**Figure 6:**
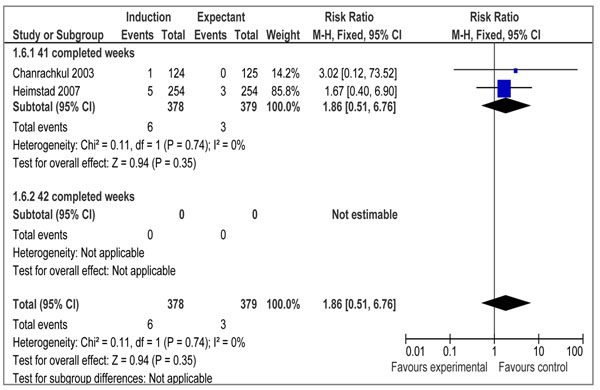
Induction of labour versus expectant management Outcome: Outcome: Birth Asphyxia

Data on morbidities associated with post-term pregnancy were presented in 10 trials [20-25,28,30,32,33] that met our eligibility criteria. We conducted meta-analyses to assess the impact of IOL at or beyond 41 weeks on meconium aspiration syndrome, birth asphyxia, and macrosomia. Our analyses demonstrated a statistically significant reduction in the incidence of meconium aspiration syndrome (RR=0.43 95% CI 0.23- 0.79) (figure 4) based on seven studies and macrosomia (RR=0.72 95% CI 0.54-0.98) (figure 5) also based on seven trials. The impact on birth asphyxia (RR=1.86 95% CI 0.51-6.76; 2 trials) (figure 6) failed to reach a level of statistical significance.

### Evidence from quasi-experimental studies

Three quasi randomized trials have been included in our study [35-37]. One study comprising of 363 women was conducted in United Kingdom over a period of 21 months. The women were allocated for either IOL (n=195) at 290 days of gestation or expectant management (n=207). The study reported one neonatal death in the induced arm and one stillbirth in the expectant arm [35]. Another prospective study was under taken from September 2000 to September 2001 in Sobhraj maternity hospital in Karachi, Pakistan [36]. This included a total of 100 prolonged uncomplicated pregnancies. Labour was induced in 50 patients at 294 days of gestation while the remaining 50 were managed expectantly. Different methods of induction were used. The choice of method depended on whether or not the cervix was favorable. The study reported a total of 9 perinatal deaths. Perinatal mortality was twice more in the controlled group (5 stillbirths and 1 neonatal death) as compared to the intervention group (2 stillbirths and 1 neonatal death). The difference in perinatal mortality between the two groups was reported as being significant (P≤ 0.05) [36]. A third study comprising of 156 uncomplicated post-date pregnancies was conducted. The study group comprised of 78 patients who were managed expectantly and the control group consisted of 78 women who were induced labour on day 294 of gestation. This study reported one neonatal death due to severe congenital heart disease in the expectant group and one neonatal death due to asphyxia in the induced group [37].

### Evidence from observational studies

Six relevant observational studies were identified [38-43]. Two of these were prospective studies. One prospective study of 395 singletons, uncomplicated post-term pregnancies was conducted at Queen Alia Military Hospital, Jordan from January 2001 to July 2002 [38]. The study comprised of two groups, one group being managed by IOL (n=175) while the other was managed expectantly (n=220). Labour was induced by inserting dinoprostone 3 mg vaginal pessaries in the posterior vaginal fornix. Continuous fetal heart rate monitoring was done in both groups. The study reported no statistically significant differences in the fetal/ neonatal outcome measures between the two groups. There were no perinatal deaths in either groups (P=0.69) [38]. Another study addressed the effect of induced labor using pitocin IV drip in decreasing the incidence of perinatal complications and perinatal mortality of prolonged pregnancy [39]. Induced labor was used in a study group comprising of 126 prolonged, nulliparous and uncomplicated pregnancies. The control group had 128 prolonged, nulliparous and uncomplicated pregnancies that were managed expectantly. The perinatal mortality was 0 in the study group and 3.1% in the control group. Also, the asphyxia rate was 8.7% in the study group and 13.3% in the control. The percentage of caesarean deliveries was the same in both groups. The results of this study suggest that induced labor is safe, effective and practical in the management of prolonged pregnancies [39].

The remaining 4 studies were retrospective studies. One study compared the outcome of expectant management of post-term pregnancy with active management in Masaryk University, Brno [41]. The study comprised of two groups. Group I included 1906 women, who were managed actively by IOL in the 41^st^ week. Group II included 2008 parturients who were managed expectantly till the end of the 42^nd^ week at which labour was induced. The study reported no differences in perinatal mortality and morbidity between the two groups and concluded that there was no need to consider the expectant management of the post-term pregnancies to be dangerous [41]. Similar conclusion was drawn by a retrospective analysis comparing the outcomes of post-term pregnancies managed actively by labour inductions beginning at 42 weeks with expectant management of labour [40]. The induced group comprised of 185 women while the group with spontaneous onset of labour had 119 women. One stillbirth occurred in both groups. There was also no statistical difference in the maternal or fetal morbidity between the two groups thus suggesting that standard clinical management is sufficient to assure optimal perinatal outcome in post-term pregnancies [40].

A study conducted in Canada assessed changes in the rates of labour induction in post-term pregnancies and its effects on the rates of stillbirth and cesarean section during1980-1995 [42]. This study demonstrated a significant increase in the rates of labour induction among women delivering at 41 or more weeks' gestation in all hospitals and provinces studied. The rate of stillbirth among deliveries at 41 or more weeks' gestation decreased significantly, from 2.8 per 1000 total births in 1980 to 0.9 per 1000 total births in 1995 (p < 0.001). The study concluded that the increased rate of labour induction at 41 or more weeks' gestation may have contributed to the decreased stillbirth rates [42]. An observational study compared the impact of IOL with spontaneous onset of labour among post term pregnancies (> or = 294 days) between July 1980 -December 1984 at Chicago Lying-In Hospital. The study comprised of 12,930 deliveries from which 707 gestations were prolonged (5.5%). Labor started spontaneously in 62%, and 38% underwent induction; the perinatal mortality was 20.5/1000 among those with spontaneous onset of labor while no deaths occurred among those in whom labour was induced. The study concluded that prolonged gestation had a high perinatal morbidity and mortality rate and "active management" (induction at 42 weeks) prevented perinatal deaths in this group thereby justifying an active approach for post-term pregnancies [43].

## Discussion

Meta-analyses of randomized controlled trials demonstrate that a policy of induction of labour for pregnancies at or beyond 41 weeks as compared to expectant management of gestation is associated with fewer perinatal deaths, but no significant difference in the rate of stillbirth. The above mentioned results are in accordance to the findings of the Cochrane review by Gulmezoglu et al 2009 [10]. This review included a total of 12 trials and reported a non-significant reduction in stillbirth risk (RR = 0.28, 95% CI: 0.05–1.67), but a statistically significant reduction in perinatal mortality (RR = 0.30, 95% CI: 0.09–0.99). An update of this Cochrane review having 14 trials reports similar results [Stillbirth: 41 complete weeks (RR = 0.29; 95% CI: 0.06 – 1.38), 42 complete weeks (not estimable); perinatal death: 41 complete weeks (RR = 0.27; 95% CI: 0.08 – 0.98), 42 complete weeks (RR = 0.41; 95% CI: 0.06 – 2.73) (Gulmezoglu M and Middleton P 2010, personal communication). The Cochrane review also included trials that compared the impact of IOL with expectant management between 37-40 completed weeks. In our review we have focused only on pregnancies at or beyond 41 weeks. This is based on the fact that most of the complications of prolonged pregnancy are known to increase beyond this gestational age [1,6,8,44,45]. Our review also includes data from quasi experimental studies and observational studies and we have used CHERG rules and GRADE criteria for selection of an appropriate estimate for inclusion in the LiST model.

Two systemic reviews by Wennerholm et al. 2009 [46] and Sanchez-Ramos et al. 2003 [5] also analyzed the impact of labour induction on stillbirths and perinatal mortality. Both reviews included a total of 13 trials. These reviews, like ours had included only those studies in their meta-analysis that reported the impacts of labour induction at or beyond 41 weeks of gestation. Neither of these two reviews, however, reported a significant difference in the rate of stillbirth or perinatal mortality between active and expectant management of post-term pregnancy. The review by Sanchez-Ramos also included the studies by Cardozo et al 1986 and Katz et al 1983, while we excluded these studies from our meta-analysis because these were both quasi-experimental studies. These studies have also been excluded from the review by Gulmezoglu et al. 2009 and Wennerholm et al. 2009 on basis of them being alternate allocation trials. We also included one study by Suikkari et al. [31] that was published as an abstract only. This study was not present in Wennerholm review but included in both Gulmezoglu and Sanchez-Ramos review. A study by Sahraoui et al. published in 2005, has been included in our review, while none of the other three reviews have included it [34]. Another recent trial by Heimstad et al. [25] published in 2007 has been included in our review and the review by Wennerholm et al 2009, but is not present in either Gulmezoglu or Sanchez-Ramos review. We have excluded any non-english article that was not translated and for which the abstract did not provide adequate data. The same approach was used by Wennerholm et al. The reviews by Sanchez Ramos et al. or Gulmezoglu et al. did not exclude studies based on this criterion. However, there were no additional studies in either of the latter reviews that were not included in our review. Hence, it is unlikely that we have missed out any clinical trial because it was published in a language other than English.

### Recommendations for LiST

Table 1 shows the qualitative assessment of overall evidence regarding induction of labor at 41 weeks and beyond. The pooled estimate from 14 randomized controlled trials showed a non-significant reduction in stillbirths [RR = 0.29, 95 % CI 0.06-1.38] and a significant reduction in perinatal mortality [RR = 0.31, 95 % CI 0.11-0.88]. We graded the overall quality of evidence for stillbirths and perinatal mortality as ‘moderate’. This assessment was based on the fact that most of the studies were conducted in developed country settings and numbers of events were relatively few in the intervention and control groups. Using CHERG rules, we recommend a reduction in perinatal mortality (i.e. 69 %) as a surrogate for reduction in stillbirths. The reason for recommending perinatal mortality as surrogate for stillbirths was based on the fact most of the studies do not report disaggregated data for stillbirths but do so for perinatal mortality. In order to further support this assumption we pooled the data for early neonatal mortality in the included studies and the results showed a non-significant (RR = 0.37; 95% CI: 0.10 – 1.38) reduction in early neonatal mortality (data not shown). We suggest that the main effect of IOL in the combined outcome of ‘perinatal mortality’ could be due to stillbirths. Further evidence of effectiveness of IOL at 41 weeks comes from the fact that this approach reduces incidence of morbidities like meconium aspiration syndrome (RR=0.43 95% CI 0.23- 0.79) and macrosomia (RR=0.72 95% CI 0.54-0.98).

**Table 1:** Quality assessment of trials of elective induction of labour versus expectant management for post-term pregnancies

	Quality Assessment					Summary of Findings		
				**Directness**		**No of events**		

**No of studies (ref)**	**Design**	**Limitations**	**Consistency**	**Generalizability to population of interest**	**Generalizability to intervention of interest**	**Intervention**	**Control**	**Relative Risk (95% CI)**

***Mortality (Stillbirth): 'MODERATE'* outcome specific quality**								

14	All RCTs; only 4 out of 14 RCTs report estimable differences in stillbirth between the two groups	Small sample size in 12 studies; result statistically insignificant; wide CI: low precision	All studies are consistent in showing a tendency towards reduction of stillbirth as an outcome i.e. the direction of effect is towards benefit	5 in developing countries; rest in developed	Yes	0	4	RR (fixed): 0.29 (0.06 - 1.38)

***Mortality (Perinatal mortality): 'MODERATE'* outcome specific quality**								

14	All RCTs; only 8 out of 14 RCTs report estimable differences in perinatal mortality between the two groups	Small sample size in 12 studies	All studies are consistent in showing a tendency towards reduction in perinatal mortality as an outcome i.e. the direction of effect is towards benefit	5 in developing countries; rest in developed	Yes	1	10	RR (fixed): 0.31 (0.11 - 0.88)

***Serious morbidity (Meconium aspiration syndrome):'MODERATE'* outcome specific quality**								

7	All RCTs	Small sample sizes in all studies	Consistent with 5/7 studies showing direction of benefit	3 out of 7 in developing countries	Yes	14	33	RR (fixed): 0.43 (0.23 - 0.79)

***Serious morbidity (Birth asphyxia): 'MODERATE'* outcome specific quality**								

2	RCTs	Small sample sizes	Both the studies show a direction towards increased risk	1/2 in developing countries	Yes	6	3	RR (fixed): 1.86 (0.51 - 6.76)

***Mild morbidity (Macrosomia): 'MODERATE'* outcome specific quality**								

7	All RCTs	Small sample sizes except in one study	6/7 studies showing direction of benefit i.e. consistent	2/7 in developing countries	Yes	279	383	RR (random): 0.72 (0.54 - 0.98)

As the number of stillbirths in the randomized trials was relatively small, we also included observational studies in our review. Although the quality of evidence derived from observational studies is generally considered poor, it is worth considering data from these studies. The, evidence from observational studies included is variable, half of the studies showed a significant difference in the rate of stillbirth and perinatal death for active versus expectant management, thereby advocating the use of routine IOL for post-term pregnancies. A large scale study conducted in Canada by Sue-A-Quan et al. [42] showed a significant reduction in the rate of stillbirths as the rates of elective IOL at or beyond 41 weeks of gestation increased (p<0.001) in the years 1980 to 1995. Another study [43] comprising of a fairly large number of cases of prolonged gestation showed that the rate of perinatal mortality was significantly higher in patients in whom spontaneous labour was awaited while no deaths were reported in the induced women. The only large scale study showing no difference in perinatal mortality between the two groups was a study by Maly et al.[41]. The remaining studies had very small sample sizes, which do not have sufficient power to detect differences in perinatal mortality and/or stillbirths.

## Conclusions

There is a well-established increased risk of adverse perinatal outcome with pregnancies that extend into the post-term period. Routine induction of labour (at or beyond 41 weeks) seems to be the likely solution for preventing perinatal morbidity and mortality associated with post term pregnancy. Data on stillbirths show no significant effect of IOL post term. Using CHERG rules we propose to use a reduction of 69% (derived from effect on perinatal mortality) in incidence of stillbirths for inclusion in the LiST model for the effect of induction of labour post term.

### Key Messages

•	Elective IOL for prolonged pregnancies at or beyond 41 leads to significant reduction in perinatal mortality [RR 0.31 95 % CI 0.11-0.88] but a non-significant reduction in stillbirths [0.29, 95 % CI 0.06-1.38].

•	There was a significant reduction of 57% in the incidence of meconium aspiration syndrome (MAS) and a non-significant impact for birth asphyxia with the use of elective IOL post term.

•	There was a significant 28% reduction in the incidence of macrosomia (RR=0.72 95% CI 0.54-0.98) with elective IOL.

•	Given the association of IOL beyond 41 weeks with reduction in perinatal deaths and assuming these are mostly due to stillbirths, we are recommending an effect size of 69% reduction of risk of stillbirth with IOL for pregnancies beyond 41 weeks gestation.

## Competing interests

The authors declare no competing interests

## Authors’ Contributions

Professor Zulfiqar A Bhutta developed the review parameters and secured support. Dr. Arwa Abbas Hussain and Dr. Mohammad Yawar Yakoob did the literature search, data extraction and analysis under the supervision of Professor Bhutta. Dr. Aamer  Imdad contributed to later revision of the manuscript. Dr. Zulfiqar A. Bhutta gave advice in all the aspects of the project and was the overall supervisor.

## Supplementary Material

Additional file 1 Characteristics of included Studies; Randomized Controlled Trials Click here for file

Additional file 2 Characteristics of included studies: Quasi-experimental trials Click here for file

Additional file 3 Characteristics of included studies: Observational studies Click here for file

Additional file 4 Data extraction sheet for studies included in the review Click here for file
