# Supplementary material for: Elective induction for pregnancies at or beyond 41 weeks of gestation and its impact on stillbirths: a systematic review with meta-analysis
Source: BMC Public Health. 2011 Apr 13;11(Suppl 3):S5. doi: 10.1186/1471-2458-11-S3-S5 (PMC3231911; doi:10.1186/1471-2458-11-S3-S5)
Supplement: Additional file 1 — Characteristics of included Studies; Randomized Controlled Trials [file 1471-2458-11-S3-S5-S1.doc]

**Additional file 1: Characteristics of included Studies; Randomized Controlled Trials**

| S# | Study, year | Country | No. of patients/ women | Definition of post-term pregnancy in days | Monitoring of the controlled group | Methods of induction | Quality grade |
| --- | --- | --- | --- | --- | --- | --- | --- |
| 1. | Augensen[19] 1987 | Norway | 409 | 290 | NST twice weekly | Oxytocin infusion and amniotomy | Moderate |
| 2. | Bergsjo[20] 1989 | China | 188 | 294 | Fetal kick counts, atropine test, ultrasound, and urinary estriol | Membrane stripping, oxytocin infusion, and amniotomy | Moderate |
| 3. | Chanrachkul[21] 2003 | Thailand | 249 | 290 | NST weekly and AFI; twice weekly after 43 wk’ gestation | Amniotomy and oxytocin infusion | Moderate |
| 4. | Dyson [22] 1987 | USA | 302 | 287 | NST twice weekly and AFI weekly between 41 and 42 wk | PGE2 gel intravaginally and oxytocin infusion with amniotomy | Moderate |
| 5. | Gelisen[23] 2005 | Turkey | 600 | 287 | NST and AFI twice weekly and BPP once 3_5 days after randomization | Misoprostol 50 mg intravaginally every 6 h_3 and amniotomy and oxytocin infusion or oxytocin infusion or Foley catheter with balloon above the internal cervical os filled with 50 ml saline and oxytocin infusion | Moderate |
| 6. | Hannah[24] 1992 | Canada | 3407 | 287 | Fetal kick counts daily; NST and AFI two to three times per week | PGE2 gel (0.5 mg) intracervically every 6 h _3 and/or oxytocin infusion, amniotomy, or both | High |
| 7. | Heimstadt [25] 2007 | Norway | 508 | 289 | AFI, and NST every third day | Misoprostol 50 ug intravaginally every 6 h or PGE2 gel (0.5 mg) intracervically very 12 h during 2 days or amniotomy and oxytocin infusion | High |
| 8. | Henry [26] 1969 | UK | 112 | 290 | Amnioscopy | Amniotomy and oxytocin infusion | Moderate |
| 9. | Herabutya [27] 1992 | Thailand | 108 | 294 | NST once weekly and twice weekly after 43 wk | PGE2 gel (0.5 mg) intracervically and/or oxytocin infusion, amniotomy, or both | Low |
| 10. | James[28] 2001 | India | 74 | 287 | Fetal kick counts daily and BPP on alternate days | Extraamniotic saline infusion if Bishop score _ 5; if _ 5, membrane stripping, amniotomy, and oxytocin infusion | Low |
| 11. | Martin[29] 1989 | USA | 22 | 287 | NST and AFI weekly | Laminaria tents and oxytocin infusion | Low |
| 12. | NICHHD[30] 1994 | USA | 440 | 287 | NST and AFI twice  weekly | PGE2 or placebo gel intracervically, oxytocin infusion, and amniotomy | Moderate |
| 13. | Ocon [32] 1997 | Spain | 113 | 294 | NST, biophysical profile and amnioscopy | PGE2 gel (0.5 mg) followed by induction of labour | Low |
| 14. | Sahraoui [34] 2005 | Tunisia | 150 | 290 | Monitored with RCF on alternate days up to 42 weeks. | Prostaglandin gel cervical ripening | Low |
| 15. | Suikkari[31] 1983 | Finland | 119 | 290 | NST, HPL, serum estriol, and AFI every 3 d | Amniotomy and oxytocin infusion | Low |
| 16. | Witter [33] 1987 | USA | 200 | 294 | Urinary estriol, creatinine ratio and OCT twice a week and thrice a week after 43 weeks | Oxytocin and amniotomy | Moderate |

NST: non stress test, AFI: amniotic fluid index
