# Supplementary material for: Elective induction for pregnancies at or beyond 41 weeks of gestation and its impact on stillbirths: a systematic review with meta-analysis
Source: BMC Public Health. 2011 Apr 13;11(Suppl 3):S5. doi: 10.1186/1471-2458-11-S3-S5 (PMC3231911; doi:10.1186/1471-2458-11-S3-S5)
Supplement: Additional file 2 — Characteristics of included studies: Quasi-experimental trials [file 1471-2458-11-S3-S5-S2.doc]

**Additional file 2**: Characteristics of included studies: Quasi-experimental trials

| S# | Study, year | Country | No. of patients/ women | Definition of post-term pregnancy in days | Monitoring of the controlled group | Methods of induction | Quality grade |
| --- | --- | --- | --- | --- | --- | --- | --- |
| 1. | Cardozo 1986 [35] | UK | 402 | 290 | Daily fetal kick  counts, NST every  other day | PGE2 gel, oxytocin  infusion, and  amniotomy | Low |
| 2. | Iqbal 2004 [36] | Pakistan | 100 | 294 | Fetal kick charting, ultrasound, AFI, and NST weekly till 42wks and twice weekly till 43 weeks | Use of balloon catheter, sweeping of membranes, amniotomy, oxytocin infusion, and prostaglandins. | Low |
| 3. | Katz 1983 [37] | Israel | 156 | 294 | Fetal kick counts  twice daily,  amnioscopy and  OCT every 3 days | Amniotomy and  oxytocin infusion | Low |
