# Supplementary material for: Elective induction for pregnancies at or beyond 41 weeks of gestation and its impact on stillbirths: a systematic review with meta-analysis
Source: BMC Public Health. 2011 Apr 13;11(Suppl 3):S5. doi: 10.1186/1471-2458-11-S3-S5 (PMC3231911; doi:10.1186/1471-2458-11-S3-S5)
Supplement: Additional file 3 — Characteristics of included studies: Observational studies [file 1471-2458-11-S3-S5-S3.doc]

**Additional file 3: Characteristics of included studies: Observational studies**

| S# | Study, year | Country | Study design | No. of patients/ women | Definition of post-term pregnancy in days | Monitoring of the controlled group | Methods of induction | Quality grade |
| --- | --- | --- | --- | --- | --- | --- | --- | --- |
| 1. | Al-Taani 2003 [38] | Jordan | Prospective cohort | 395 | 289-295 | Fetal heart rate monitoring | cervical priming, with dinoprostone 3mg vaginal pessaries, amniotomy and oxytocin augmentation | Very low |
| 2. | Bian 1990 [39] | China | Prospective study | 254 | Not given in the abstract | Not mentioned in the abstract | IV pitocin | Very low |
| 3. | Hauth 1980 [40] | Not given in abstract | Retrospective comparative study | 304 | Not defined in days; induction starting at 42 weeks | Not mentioned in abstract | Not mentioned in abstract | Very low |
| 4. | Malý2002 [41] | Brno | Retrospective study | 3914 | Not defined in days; induction in the 41^st^ week | Not mentioned in abstract | Not mentioned in abstract | Very low |
| 5. | Sue-A-Quan 1999 [42] | Canada | Retrospective comparative study; comparison of rates | Rates not given | Not defined; rates assessed at 40, 41 & 42 weeks | Not mentioned | Not mentioned | Very low |
| 6. | Votta 1993[43] | USA | Retrospective Observational study | 707 | 294 | Not mentioned in the abstract | Not mentioned in the abstract | Very low |
